# Supplementary material for: Warming and trophic structure tightly control phytoplankton bloom amplitude, composition and succession
Source: PLoS One. 2024 Oct 4;19(10):e0308505. doi: 10.1371/journal.pone.0308505 (PMC11451980; doi:10.1371/journal.pone.0308505)
Supplement: S1 File — S1 Fig. Median Chl a concentrations in the different treatments during pre-bloom (A), bloom (B), and post-bloom periods (C). Blue indicates the control treatment (C), red, green, and yellow indicated the heated (T), mesozooplankton exclusion (MicroZ), and heated and mesozooplankton exclusion (TMicroZ) treatments, respectively. Significance level of RM-ANOVAs: * = p-value < 0.05; ** = p-value < 0.01; *** = p-value < 0.001. The letters indicate significant differences between treatments based on post hoc pairwise tests. Boxplots that share the same letter are not significantly different. Boxplots with different letters differ are significantly different (p-value < 0.05). Nonsignificant RM-ANOVAs has no stars or letters. S2 Fig. Median nutrient concentrations in the mesocosms between the different treatments for pre-bloom, bloom, and post-bloom periods. Nutrients are NH4+ (A, B, and C), NO2- (D, E, and F), NO3- (G, H, and I), SiO2 (J, K, and L), and PO43- (M, N and O). Blue, control treatment (C); red, green, and yellow, the heated (T), mesozooplankton exclusion (MicroZ), and heated and mesozooplankton exclusion (TMicroZ) treatments, respectively. Significance level of RM-ANOVAs: * = p-value < 0.05; ** = p-value < 0.01; *** = p-value < 0.001. The letters indicate significant differences between treatments based on post hoc pairwise tests. Boxplots that share the same letter are not significantly different. When the letters differ, they are significantly different (p-value < 0.05). A lack of asterisks and letters indicates RM-ANOVAs was nonsignificant. S3 Fig. Median concentrations of taxonomic pigments in the different treatments during pre-bloom, bloom, and post-bloom periods: Chl b (A, B, and C), Prasinoxanthin (D, E, and F), Zeaxanthin (G, H, and I), Alloxanthin (J, K, and L), 19HF (M, N, and O), Fucoxanthin (P, Q, and R), and Peridinin (S, T, and U). Blue, control (C); red, green, and yellow, heated (T), mesozooplankton exclusion (MicroZ), and heated and mesozoopla [file pone.0308505.s001.docx]

Supporting Information

# Supporting Figures and Tables

## Supporting Figures

**
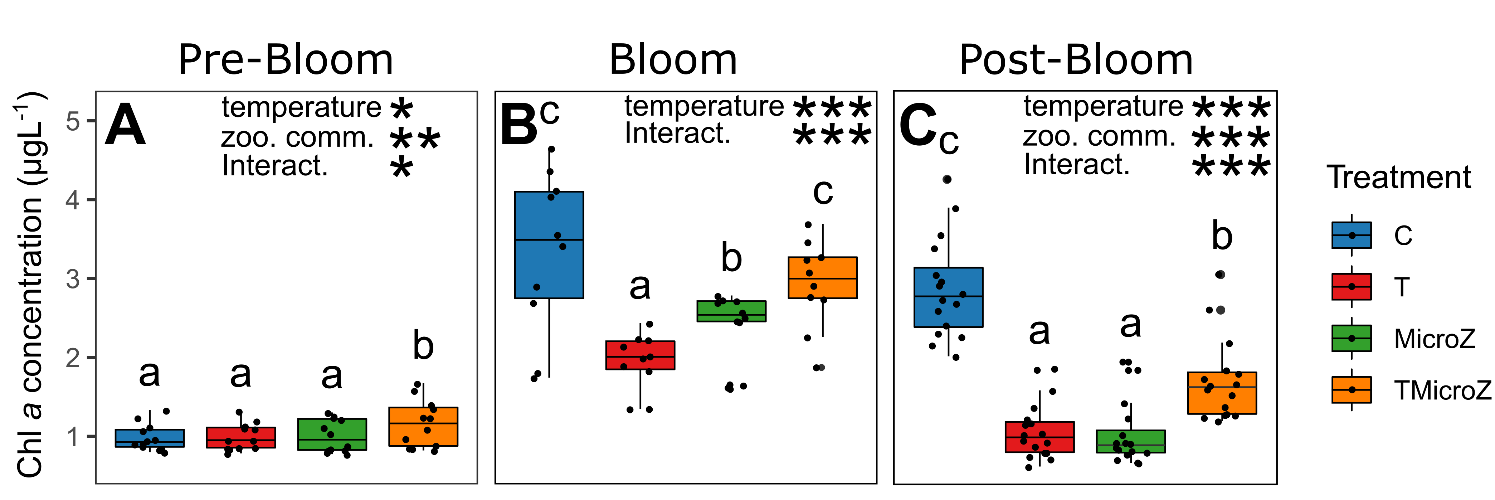
**

**Supporting Figure S1.** Median Chl *a* concentrations in the different treatments during pre-bloom (A), bloom (B), and post-bloom periods (C). Blue indicates the control treatment (C), red, green, and yellow indicated the heated (T), mesozooplankton exclusion (MicroZ), and heated and mesozooplankton exclusion (TMicroZ) treatments, respectively. Significance level of RM-ANOVAs: * = *p*-value < 0.05; ** = *p*-value < 0.01; *** = *p*-value < 0.001. The letters indicate significant differences between treatments based on post hoc pairwise tests. Boxplots that share the same letter are not significantly different. Boxplots with different letters differ are significantly different (*p-*value < 0.05). Nonsignificant RM-ANOVAs have no stars or letters.


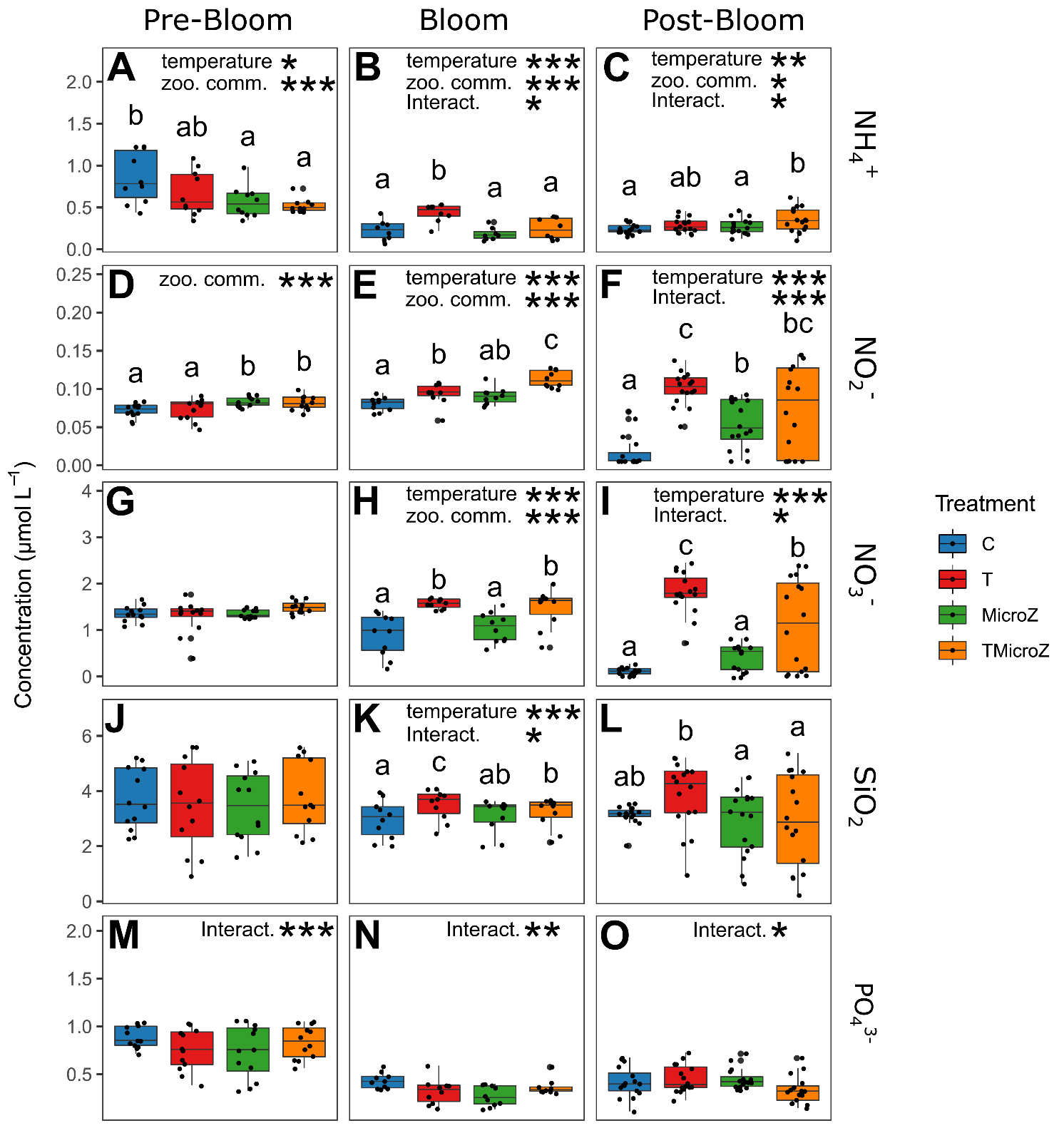


**Supporting Figure S2.** Median nutrient concentrations in the mesocosms between the different treatments for pre-bloom, bloom, and post-bloom periods. Nutrients are NH_4_^+^ (A, B, and C), NO_2_^-^ (D, E, and F), NO_3_^-^ (G, H, and I), SiO_2_ (J, K, and L), and PO_4_^3-^ (M, N and O). Blue, control treatment (C); red, green, and yellow, the heated (T), mesozooplankton exclusion (MicroZ), and heated and mesozooplankton exclusion (TMicroZ) treatments, respectively. Significance level of RM-ANOVAs: * = *p*-value < 0.05; ** = *p*-value < 0.01; *** = *p*-value < 0.001. The letters indicate significant differences between treatments based on post hoc pairwise tests. Boxplots that share the same letter are not significantly different. When the letters differ, they are significantly different (*p-*value < 0.05). A lack of asterisks and letters indicates RM-ANOVAs were nonsignificant.

**
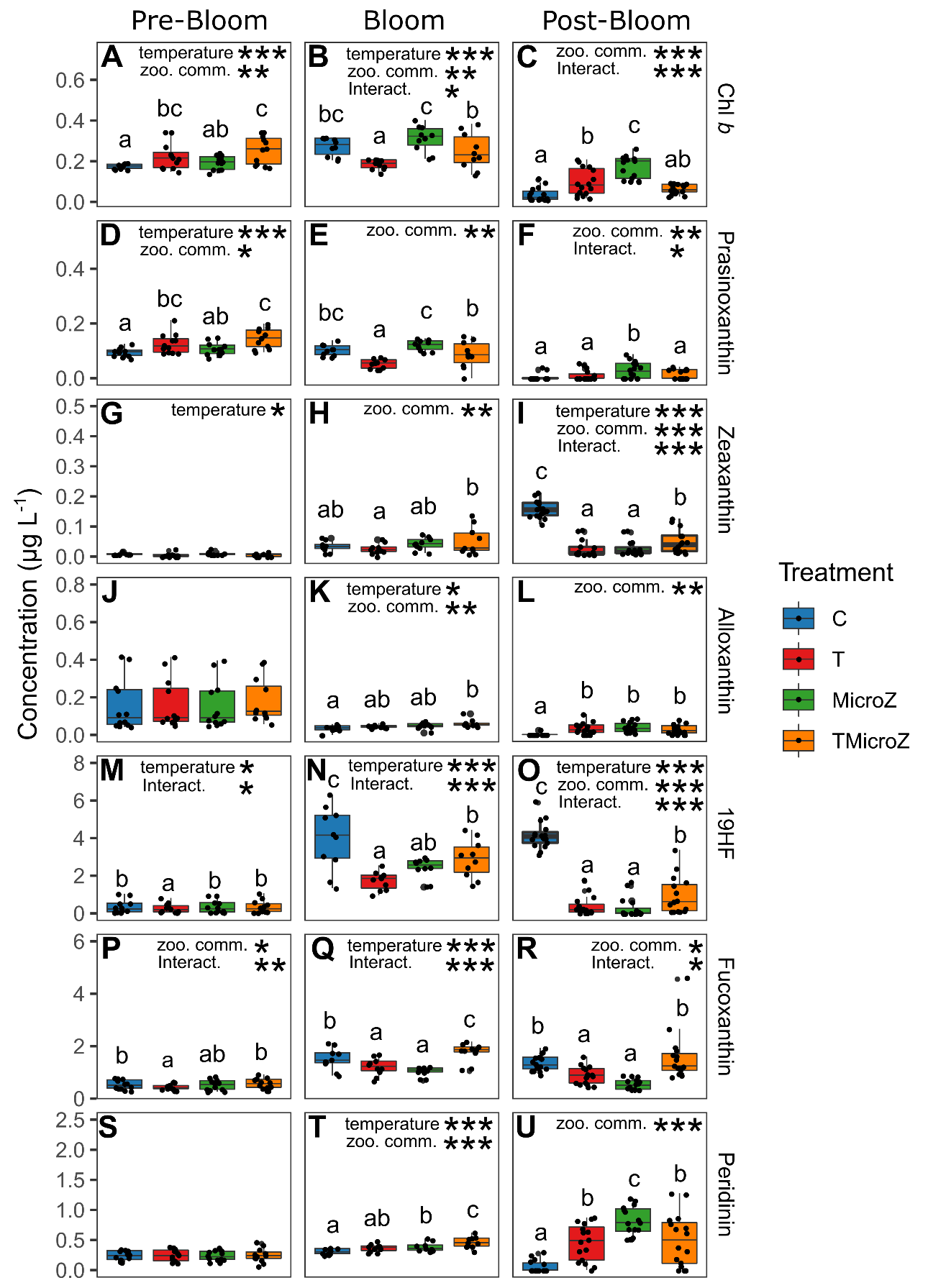
**

**Supporting Figure S3.** Median concentrations of taxonomic pigments in the different treatments during pre-bloom, bloom, and post-bloom periods: Chl *b* (A, B, and C), Prasinoxanthin (D, E, and F), Zeaxanthin (G, H, and I), Alloxanthin (J, K, and L), 19HF (M, N, and O), Fucoxanthin (P, Q, and R), and Peridinin (S, T, and U). Blue, control (C); red, green, and yellow, heated (T), mesozooplankton exclusion (MicroZ), and heated and mesozooplankton exclusion (TMicroZ) treatments, respectively. Significance level of RM-ANOVAs: * = *p*-value < 0.05; ** = *p*-value < 0.01; *** = *p*-value < 0.001. The letters indicate significant differences between treatments based on post-hoc pairwise tests. Boxplots that share the same letter are not significantly different. When the letters differ, they are significantly different (*p-*value < 0.05). Nonsignificant RM-ANOVAs have no stars or letters.


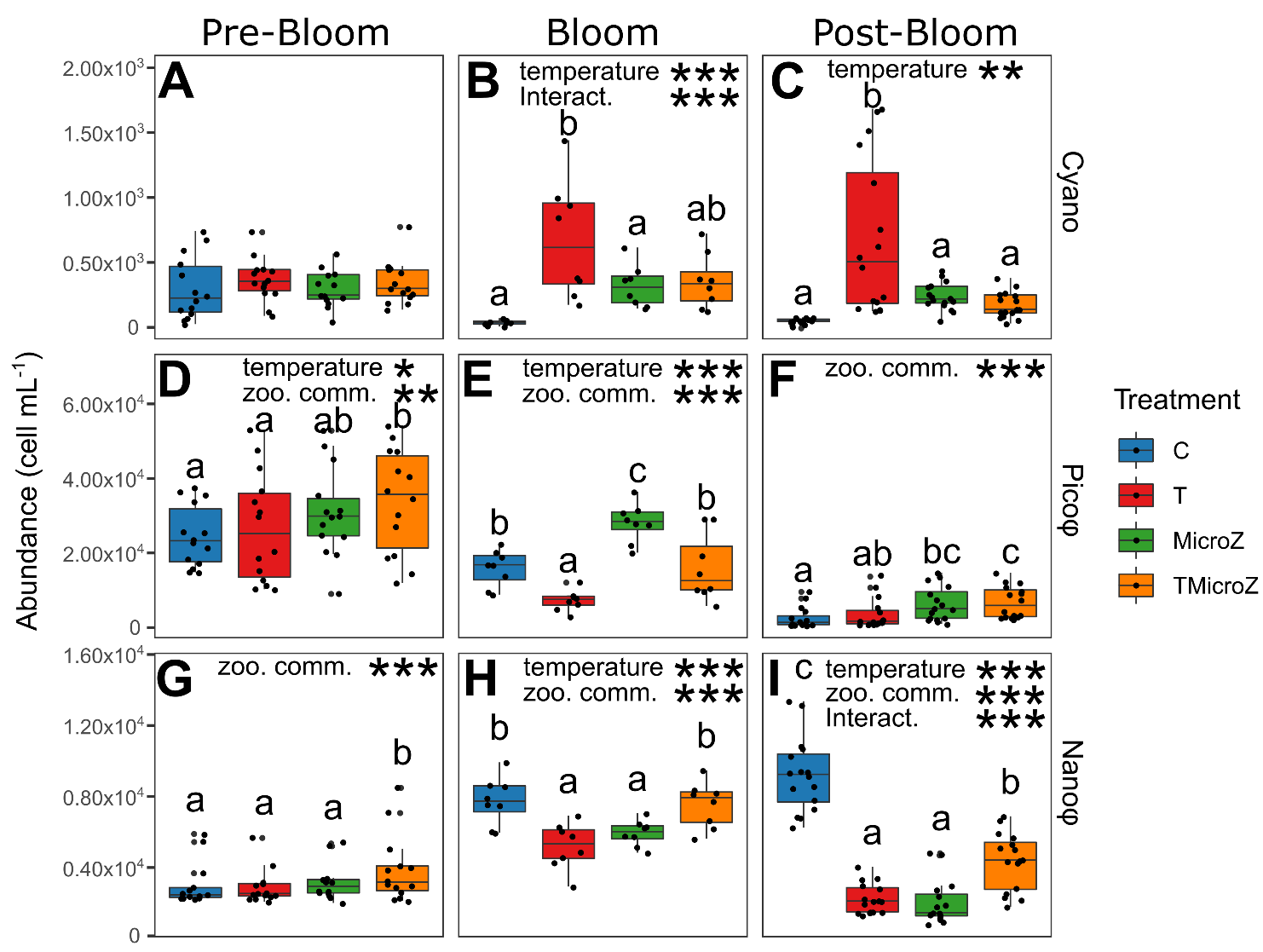


**Supporting Figure S4.** Median abundance of phytoplanktonic groups in the different treatments during pre-bloom, bloom, and post-bloom periods: Cyano (A, B, and C), Picoφ (D, E, and F), and Nanoφ (G, H, and I). Blue, control (C); red, green, and yellow, heated (T), mesozooplankton exclusion (MicroZ), and heated and mesozooplankton exclusion (TMicroZ) treatments, respectively. Significance level of RM-ANOVAs: * = *p*-value < 0.05; ** = *p*-value < 0.01; *** = *p*-value < 0.001. The letters indicate significant differences between treatments based on post hoc pairwise tests. Boxplots that share the same letter are not significantly different. When the letters differ, they are significantly different (*p-*value < 0.05). Nonsignificant RM-ANOVAs have neither stars nor letters.


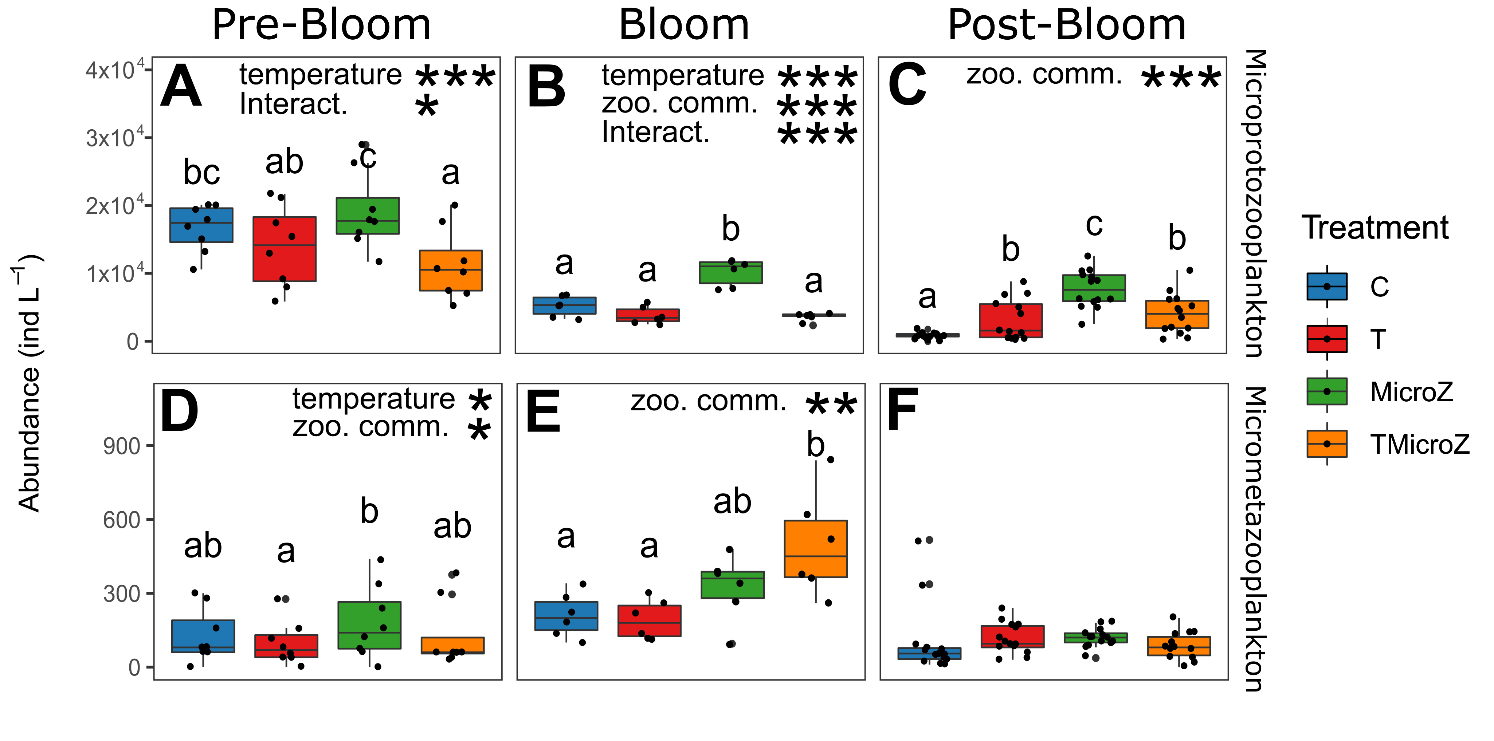


**Supporting Figure S5.** Median abundance of protozooplankton (A, B and C) and micrometazooplankton (D, E and F) in the different treatments during pre-bloom, bloom, and post-bloom periods. Blue, control (C); red, green, and yellow, heated (T), mesozooplankton exclusion (MicroZ), and heated and mesozooplankton exclusion (TMicroZ) treatments, respectively. Significance level of RM-ANOVAs: * = *p*-value < 0.05; ** = *p*-value < 0.01; *** = *p*-value < 0.001. The letters indicate significant differences between treatments based on post hoc pairwise tests. Boxplots that share the same letter are not significantly different. When the letters differ, they are significantly different (*p-*value < 0.05). Nonsignificant RM-ANOVAs have neither stars nor letters.


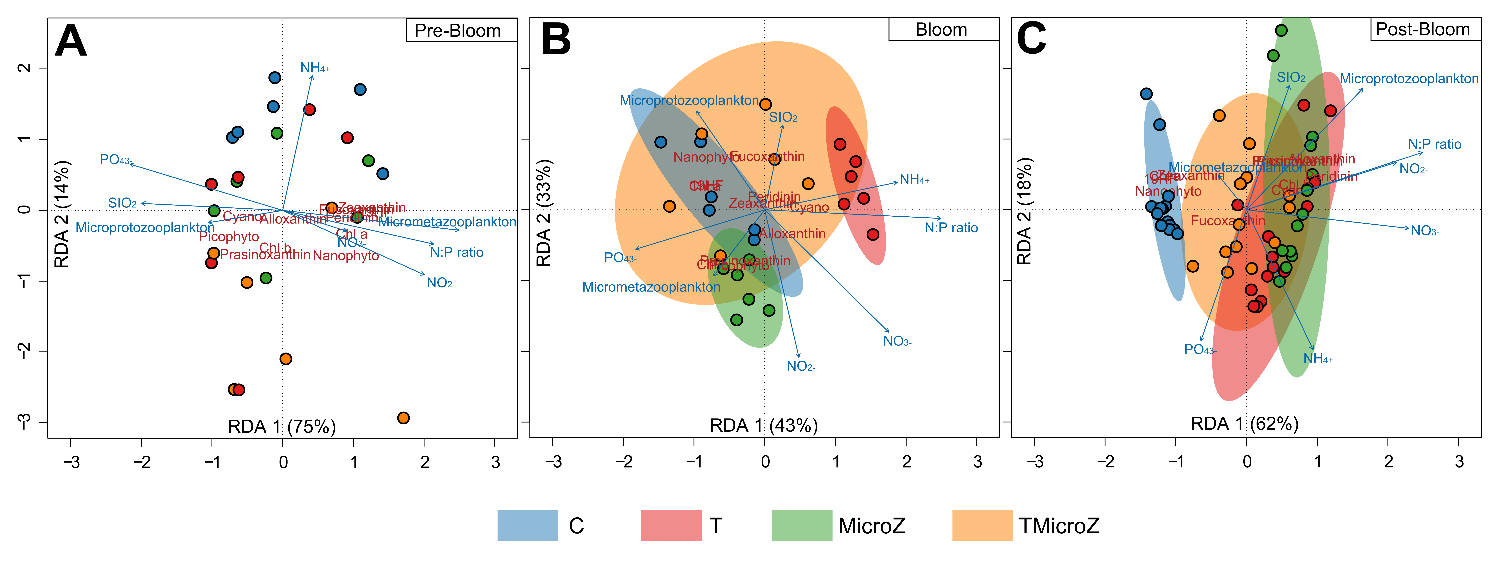


**Supporting Figure S6.** Redundancy Analysis (RDA) between phytoplankton composition (pigment and cytometry and environmental parameters. (A) Pre-bloom period, (B) Bloom period, and (C) Post-Bloom periods. Dots present sampling dates community composition. Blue arrows present the projections of environmental parameters. Red labels present projection of the pigments and cytometry groups. The proximity between environmental parameters or pigments and cytometry groups and sampling dates indicates characteristic associations. Ellipses represents 95% confidence distribution interval of community for each treatment (C, T, MicroZ and TMicroZ). Ellipses were not represented for Pre-bloom (A) because they were overlapping and communities showed no significant differences.

## Supporting Tables

**Supporting Table S1**. Light penetration mean in the mesocosms and diffuse attenuation coefficient (Kd).

|  | PAR (µmol quanta m^-2^ s^-1^) | | | Kd (m^-1^) |
| --- | --- | --- | --- | --- |
| Experimental Day | Above surface | Below surface | 1m depth |  |
| Day 5 | 244±120 | 179±99 | 78±56 | 0.94±0.40 |
| Day 12 | 464±340 | 236±113 | 135±102 | 0.67±0.43 |
| Day 13 | 417±304 | 284±108 | 179±66 | 0.45±0.29 |
| Day 14 | 439±230 | 287±84 | 156±84 | 0.69±0.56 |
| Day 15 | 537±329 | 305±99 | 142±105 | 1.00±0.79 |
| Day 16 | 513±306 | 342±166 | 137±107 | 1.07±0.59 |
| Day 17 | 514±225 | 367±177 | 139±76 | 1.02±0.34 |

**Supporting Table S2**. Statistical results for nutrients (NH_4_^+^, NO_2_^-^, NO_3_^-^, Si, PO_4_^3-^) concentration differences between treatments on two factors (Temperature x Zooplankton community ­­[Zoo. Comm]), for Pre-Bloom, Bloom and Post-Bloom periods. Bold values indicate significant tests (p-values < 0.05).

|  |  | Pre-bloom | | | | Bloom | | | | Post-bloom | | | |
| --- | --- | --- | --- | --- | --- | --- | --- | --- | --- | --- | --- | --- | --- |
|  | Factor | Test | df | Test value | p-value | Test | df | Test value | p-value | Test | df | Test value | p-value |
| NH_4_^+^ | Temperature | RM-ANOVA | 32 | **4,16** | **0,0498** | RM-ANOVA | 25 | **21,71** | **0,0001** | RM-ANOVA | 53 | **9,24** | **0,0037** |
|  | Zoo. Comm. |  |  | **14,74** | **0,0005** |  |  | **16,66** | **0,0004** |  |  | **6,16** | **0,0163** |
|  | Interact |  |  | 1,52 | 0,227 |  |  | 6,35 | **0,0185** |  |  | 0,56 | 0,4571 |
| NO_2_^-^ | Temperature | RM-ANOVA | 39 | 0,0213 | 0,8846 | RM-ANOVA | 32 | **23,25** | **<0,0001** | RM-ANOVA | 53 | **29,9** | **0,0001** |
|  | Zoo. Comm. |  |  | **22,24** | **0,0001** |  |  | **15,92** | **0,0004** |  |  | 1,35 | 0,251 |
|  | Interact |  |  | 0,56 | 0,457 |  |  | 1,207 | 0,2802 |  |  | **30,89** | **<0,0001** |
| NO_3_^-^ | Temperature | RM-ANOVA | 39 | 0,4601 | 0,5016 | RM-ANOVA | 32 | **41,08** | **<0,0001** | Kruskall-Wallis | 1 | **23,43** | **<0,0001** |
|  | Zoo. Comm. |  |  | 3,1397 | 0,0842 |  |  | 0,21 | 0,6462 |  |  | 0,21 | 0,64 |
|  | Interact |  |  | 3,1347 | 0,0842 |  |  | 2,31 | 0,1385 |  |  | NA | NA |
| Si | Temperature | RM-ANOVA | 39 | 0,07 | 0,79 | RM-ANOVA | 32 | **29,04** | **<0,0001** | Kruskall-Wallis | 1 | 2,34 | 0,1258 |
|  | Zoo. Comm. |  |  | 0,000191 | 0,97 |  |  | 2,08 | 0,1588 |  |  | 1,63 | 0,2021 |
|  | Interact |  |  | 1,36537 | 0,25 |  |  | **10,44** | **0,029** |  |  | NA | NA |
| PO_4_^3-^ | Temperature | RM-ANOVA | 39 | 0,26 | 0,6149 | RM-ANOVA | 32 | 0,08565 | 0,7717 | RM-ANOVA | 53 | 1,37 | 0,2473 |
|  | Zoo. Comm. |  |  | 1,44 | 0,2372 |  |  | 3,7 | 0,0632 |  |  | 1,53 | 0,2212 |
|  | Interact |  |  | **15,74** | **0,0003** |  |  | **11,29** | **0,002** |  |  | **4,96** | **0,0303** |

|  |  | Pre-bloom | | | | Bloom | | | | Post-bloom | | | |
| --- | --- | --- | --- | --- | --- | --- | --- | --- | --- | --- | --- | --- | --- |
|  | Factor | Test | df | Test value | p-value | Test | df | Test value | p-value | Test | df | Test value | p-value |
| Chl *b* | Temperature | RM-ANOVA | 39 | **31,4** | **<0,0001** | RM-ANOVA | 32 | **23,25** | **<0,0001** | RM-ANOVA | 53 | 2,32 | 0,1341 |
|  | Zoo. Comm. |  |  | **7,55** | **0,009** |  |  | **11,09** | **0,0022** |  |  | **28,95** | **<0,0001** |
|  | Interact |  |  | 0,067 | 0,7971 |  |  | **0,62** | **0,04356** |  |  | **65,91** | **<0,0001** |
| Prasi | Temperature | RM-ANOVA | 39 | **29,99** | **<0,0001** | RM-ANOVA | 32 | **27,78** | **<0,0001** | RM-ANOVA | 53 | 0,43 | 0,5129 |
|  | Zoo. Comm. |  |  | **5,95** | **0,0194** |  |  | **10,86** | **0,0024** |  |  | **11,91** | **0,0011** |
|  | Interact |  |  | 0,24 | 0,6267 |  |  | 1,08 | 0,3056 |  |  | **5,54** | **0,0223** |
| Zea | Temperature | RM-ANOVA | 39 | **11,33** | **0,0017** | RM-ANOVA | 32 | 0,0057 | 0,9401 | RM-ANOVA | 53 | **141,88** | **<0,0001** |
|  | Zoo. Comm. |  |  | 0,22 | 0,6415 |  |  | **10,19** | **0,0032** |  |  | **131,12** | **<0,0001** |
|  | Interact |  |  | 0,052 | 0,8209 |  |  | 2,54 | 0,1211 |  |  | **274,81** | **<0,0001** |
| Allo | Temperature | RM-ANOVA | 39 | 8,6 | 0,056 | RM-ANOVA | 32 | **6,34** | **0,017** | Kruskall-Wallis | 1 | 3,19 | 0,07387 |
|  | Zoo. Comm. |  |  | 2,58 | 0,1159 |  |  | **11,35** | **0,002** |  |  | **9,4** | **0,002168** |
|  | Interact |  |  | 3,98 | 0,053 |  |  | 0,015 | 0,9037 |  |  | NA | NA |
| 19HF | Temperature | RM-ANOVA | 39 | **4,62** | **0,0378** | RM-ANOVA | 32 | **29,56** | **<0,0001** | RM-ANOVA | 53 | **229,92** | **<0,0001** |
|  | Zoo. Comm. |  |  | 3,4 | 0,0729 |  |  | 0,49 | 0,4895 |  |  | **258,51** | **<0,0001** |
|  | Interact |  |  | **4,42** | **0,042** |  |  | **33,48** | **<0,0001** |  |  | **457,72** | **<0,0001** |
| Fuco | Temperature | RM-ANOVA | 39 | 0,52 | 0,48 | RM-ANOVA | 32 | **15,51** | **0,0004** | RM-ANOVA | 53 | **5,24** | **0,0261** |
|  | Zoo. Comm. |  |  | **4,75** | **0,0353** |  |  | 0,37 | 0,5476 |  |  | 0,18 | 0,6768 |
|  | Interact |  |  | **10,43** | **0,0025** |  |  | **83,07** | **<0,0001** |  |  | **37,08** | **<0,0001** |
| Peri | Temperature | RM-ANOVA | 39 | 0,55 | 0,4646 | RM-ANOVA | 32 | **13,36** | **0,0009** | Kruskall-Wallis | 1 | 0,61 | 0,4331 |
|  | Zoo. Comm. |  |  | 0,013 | 0,9083 |  |  | **19,53** | **0,00001** |  |  | **15,58** | **<0,0001** |
|  | Interact |  |  | 0,19 | 0,669 |  |  | 0,23 | 0,635 |  |  | NA | NA |

**Supporting Table S3**. Statistical results for Pigments (Chl *b*, Prasi, Zea, Allo, 19HF, Fuco, Peri) concentration differences between treatments on two factors (Temperature x Zooplankton community ­­[Zoo. Comm]), for Pre-Bloom, Bloom and Post-Bloom periods. Bold values indicate significant tests (p-values < 0.05).

| Family | Genus/sp | Pre-Bloom | | | | | | | | Bloom | | | | Post-Bloom | | | | | | | |
| --- | --- | --- | --- | --- | --- | --- | --- | --- | --- | --- | --- | --- | --- | --- | --- | --- | --- | --- | --- | --- | --- |
|  |  | Day 0 | | | | Day 3 | | | | Day 9 | | | | Day 14 | | | | Day 18 | | | |
|  |  | C | T | MicroZ | TMicroZ | C | T | MicroZ | TMicroZ | C | T | MicroZ | TMicroZ | C | T | MicroZ | TMicroZ | C | T | MicroZ | TMicroZ |
| Chlorophyceae | undiferentiated | 89.73 ± 15.24 | 165.07 ± 19.47 | 182.3 ± 27.64 | 80.55 ± 14.26 | 118.51 ± 28.78 | 57.56 ± 22.01 | 166.76 ± 24.55 | 129.52 ± 12.7 | 102.01 ± 6.35 | 102.43 ± 5.93 | 158.3 ± 14.39 | 167.61 ± 15.24 | 172.14 ± 17.48 | 50.79 ± 3.39 | 92.52 ± 40.04 | 93.96 ± 29.63 | 99.47 ± 34.28 | 39.3 ± 33.5 | 71.95 ± 33.01 | 69.41 ± 11.85 |
| Cryptophyceae | *Teleaulax acuta* | 197.24 ± 2.54 | 205.7 ± 56.72 | 231.1 ± 43.17 | 127.82 ± 5.93 | 103.28 ± 8.47 | 87.4 ± 23.07 | 167.61 ± 23.7 | 136.29 ± 16.08 | 30.56 ± 13.63 | 50.34 ± 22.46 | 246.34 ± 44.87 | 79.17 ± 29.19 | 46.15 ± 3.83 | 67.72 ± 37.25 | 202.32 ± 105.81 | 21.16 ± 4.23 | 26.67 ± 5.5 | 125.28 ± 110.05 | 271.73 ± 237.87 | 21.16 ± 14.39 |
| Prymnesiophyceae | undiferentiated | 140.52 ± 23.7 | 70.26 ± 31.32 | 44.87 ± 9.31 | 7.13 ± 1.34 | 22.86 ± 14.39 | 20.78 ± 2.16 | 38.56 ± 15.62 | 28.78 ± 8.47 | 84.23 ± 2.96 | 99.89 ± 6.77 | 173.54 ± 26.24 | 190.47 ± 22.86 | 277.05 ± 26.48 | 27.17 ± 17.02 | 5.08 ± 1.69 | 42.33 ± 32.17 | 47.4 ± 10.16 | 22.01 ± 10.16 | 0 ± 0 | 17.78 ± 0.85 |
| Euglenophyceae | *Eutreptiella braarudii* | 0.85 ± 0.85 | 0.42 ± 0.42 | 0 ± 0 | 0 ± 0 | 16.3 ± 2.33 | 10.56 ± 2.14 | 18.62 ± 9.73 | 13.54 ± 1.69 | 22.64 ± 3.17 | 11.43 ± 1.27 | 11.64 ± 1.48 | 16.51 ± 2.96 | 25.4 ± 2.12 | 29.2 ± 23.28 | 12.7 ± 2.54 | 12.91 ± 2.33 | 19.68 ± 0.21 | 6.56 ± 5.29 | 10.58 ± 0.85 | 8.04 ± 0.42 |
| Dinophyceae | *Gymnodinium* sp. | 4.59 ± 1.2 | 8.04 ± 2.54 | 8.04 ± 1.27 | 19.26 ± 14.6 | 22.64 ± 5.29 | 6.98 ± 0.63 | 21.37 ± 3.17 | 35.34 ± 15.45 | 51.21 ± 2.96 | 42.11 ± 10.79 | 46.98 ± 7.2 | 58.62 ± 10.37 | 17.78 ± 7.62 | 144.23 ± 93.02 | 180.52 ± 10.79 | 113.06 ± 10.53 | 6.98 ± 0.21 | 19.47 ± 10.16 | 60.16 ± 7.56 | 14.6 ± 1.06 |
|  | *Prorocentrum minimum* | 13.33 ± 3.17 | 11.47 ± 0 | 16.08 ± 0.42 | 8.44 ± 0.02 | 27.51 ± 1.27 | 22.43 ± 2.12 | 42.54 ± 7.41 | 28.99 ± 2.75 | 31.74 ± 2.96 | 24.55 ± 4.23 | 43.17 ± 7.62 | 27.3 ± 11.64 | 9.73 ± 0 | 4.66 ± 4.66 | 18.41 ± 5.29 | 2.33 ± 1.06 | 1.9 ± 0.63 | 0.63 ± 0.63 | 4.87 ± 1.06 | 0.85 ± 0.85 |
| Diatomophyceae | *Chaetoceros* spp. | 32.31 ± 8.32 | 20.1 ± 3.6 | 43.53 ± 8.4 | 30.17 ± 10.84 | 22.62 ± 6.11 | 22.22 ± 8.68 | 74.54 ± 8.93 | 71.57 ± 14.01 | 51.68 ± 6.9 | 26.45 ± 9.52 | 13.41 ± 0 | 32.97 ± 7.24 | 18.83 ± 5.71 | 28.76 ± 18.65 | 5.93 ± 5.93 | 32.17 ± 9.73 | 10.3 ± 4.51 | 9.1 ± 9.1 | 0.85 ± 0.85 | 33.23 ± 17.14 |
|  | *Guinardia* spp. | 5.93 ± 4.23 | 1.9 ± 1.9 | 0 ± 0 | 1.69 ± 1.69 | 2.33 ± 2.33 | 0.85 ± 0.85 | 0.63 ± 0.63 | 1.06 ± 0.21 | 2.9 ± 2.9 | 4.87 ± 2.75 | 13.76 ± 0.21 | 1.69 ± 0.85 | 3.81 ± 2.96 | 26.24 ± 26.24 | 45.29 ± 24.13 | 1.9 ± 0.21 | 0.42 ± 0.42 | 99.68 ± 99.68 | 68.42 ± 55.72 | 0 ± 0 |
|  | *Cyclotella* sp. | 22.43 ± 1.27 | 9.31 ± 0.42 | 34.94 ± 4.42 | 32.65 ± 0.49 | 52.06 ± 7.62 | 20.53 ± 0.21 | 57.14 ± 6.77 | 46.58 ± 3.4 | 56.93 ± 0.63 | 24.76 ± 4.87 | 50.37 ± 0.42 | 15.87 ± 1.48 | 20.32 ± 2.96 | 29.84 ± 22.64 | 15.45 ± 4.87 | 17.57 ± 1.9 | 9.95 ± 2.75 | 15.87 ± 4.44 | 4.66 ± 1.69 | 14.81 ± 3.39 |
|  | *Licmophora* sp. | 9.03 ± 5.65 | 3.17 ± 1.9 | 3.39 ± 0 | 5.71 ± 0.63 | 3.17 ± 0.63 | 1.69 ± 0 | 2.12 ± 0.42 | 1.69 ± 0.85 | 8.65 ± 1.88 | 12.06 ± 1.9 | 8.47 ± 0.85 | 10.58 ± 1.69 | 9.73 ± 3.81 | 8.4 ± 5.86 | 13.9 ± 2.05 | 2.96 ± 0.42 | 7.2 ± 0.85 | 4.66 ± 2.12 | 37.86 ± 5.74 | 5.93 ± 1.69 |
|  | *Pseudo-nitzschia* spp*.* | 5.93 ± 4.23 | 1.06 ± 1.06 | 2.96 ± 1.27 | 2.54 ± 2.54 | 0.85 ± 0.85 | 0.42 ± 0.42 | 1.9 ± 0.21 | 1.27 ± 1.27 | 0 ± 0 | 2.54 ± 2.54 | 2.33 ± 0.63 | 3.81 ± 3.81 | 2.96 ± 0.42 | 3.39 ± 0 | 0 ± 0 | 4.66 ± 1.27 | 4.23 ± 4.23 | 0 ± 0 | 0 ± 0 | 10.79 ± 2.75 |

**Supporting Table S4.** Dominant phytoplankton mean abundance (±range) identified using Microscopy in each treatment (in cell mL^-1^).

|  |  | Pre-bloom | | | | Bloom | | | | Post-bloom | | | |
| --- | --- | --- | --- | --- | --- | --- | --- | --- | --- | --- | --- | --- | --- |
|  | Factor | Test | df | Test value | p-value | Test | df | Test value | p-value | Test | df | Test value | p-value |
| Cyano | Temperature | RM-ANOVA | 38 | 1,1 | 0,3007 | RM-ANOVA | 32 | **34,82** | **<0,0001** | Kruskall-Wallis | 1 | **10,47** | **0,00121** |
|  | Zoo. Comm. |  |  | 0,43 | 0,5168 |  |  | 7,69 | 0,092 |  |  | 1,87 | 0,1708 |
|  | Interact |  |  | 0,37 | 0,5439 |  |  | **23,99** | **<0,0001** |  |  | NA | NA |
| Pico | Temperature | RM-ANOVA | 39 | **5,64** | **0,0225** | RM-ANOVA | 32 | **30,82** | **<0,0001** | RM-ANOVA | 53 | 0,8 | 0,3766 |
|  | Zoo. Comm. |  |  | **9,73** | **0,0034** |  |  | **36,72** | **<0,0001** |  |  | **16,8** | **0,0001** |
|  | Interact |  |  | 0,029 | 0,8646 |  |  | 0,03 | 0,8641 |  |  | 0,71 | 0,7909 |
| Nano | Temperature | RM-ANOVA | 39 | 2,65 | 0,1117 | RM-ANOVA | 32 | 0,4826 | 0,4923 | RM-ANOVA | 53 | **46,7** | **<0,0001** |
|  | Zoo. Comm. |  |  | **12,05** | **0,0013** |  |  | 2,3781 | 0,1329 |  |  | **55,86** | **<0,0001** |
|  | Interact |  |  | 2,32 | 0,1359 |  |  | **42,88** | **<0,0001** |  |  | **187,55** | **<0,0001** |

**Supporting Table S5**. Statistical results for Pico- and Nano-phytoplankton (Cyano, Pico and Nano) concentration differences between treatments on two factors (Temperature x Zooplankton community ­­[Zoo. Comm]), for Pre-Bloom, Bloom and Post-Bloom periods. Bold values indicate significant tests (p-values < 0.05).

**Supporting Table S6.** Dominant zooplankton taxa mean abundance (±range) identified using stereomicroscope in each treatment (in ind L^-1^).

| Type | Taxa | Pre-Bloom | | | | Bloom | | | | Post-Bloom | | | | | | | |
| --- | --- | --- | --- | --- | --- | --- | --- | --- | --- | --- | --- | --- | --- | --- | --- | --- | --- |
|  |  | Day 0 | | | | Day 7 | | | | Day 14 | | | | Day 18 | | | |
|  |  | C | T | MicroZ | TMicroZ | C | T | MicroZ | TMicroZ | C | T | MicroZ | TMicroZ | C | T | MicroZ | TMicroZ |
| Copepoda | *Oithona* sp. | 0.25 | 0.12 | 0 | 0.04 ± 0.04 | 0 ± 0 | 0 ± 0 | 0 ± 0 | 0.38 ± 0.12 | 0.48 ± 0.48 | 0.35 ± 0.05 | 1.35 ± 0.85 | 0.22 ± 0.02 | 0 ± 0 | 0.2 ± 0.2 | 0.6 ± 0.2 | 0.05 ± 0.05 |
|  | *Acartia* sp. | 5.25 | 1.5 | 4.62 | 3.76 ± 1.24 | 6.38 ± 2.12 | 3.25 ± 2 | 6.25 ± 1 | 1.62 ± 0.88 | 0.88 ± 0.43 | 1.25 ± 1.05 | 5.1 ± 1 | 0.32 ± 0.08 | 0.28 ± 0.28 | 2.62 ± 2.58 | 4.3 ± 1.9 | 0.4 ± 0.1 |
|  | Oncaeidae und | 0 | 0 | 0 | 0 ± 0 | 0.11 ± 0.01 | 0 ± 0 | 0 ± 0 | 0 ± 0 | 0 ± 0 | 0.2 ± 0 | 0.45 ± 0.05 | 0.22 ± 0.07 | 0.03 ± 0.03 | 0.05 ± 0.05 | 0.5 ± 0.1 | 0.32 ± 0.02 |
|  | Nauplii | 26.5 | 12.25 | 36.5 | 23.9 ± 10.1 | 3.49 ± 1.61 | 5.38 ± 2.88 | 13.75 ± 1.75 | 5.75 ± 0 | 4.6 ± 1.25 | 18.8 ± 15.2 | 14.35 ± 0.25 | 4.4 ± 0.55 | 1.73 ± 0.22 | 70.85 ± 65.75 | 82.3 ± 31.5 | 8.05 ± 1.15 |
| Rotifera | undifferentiated | 0 | 0 | 0 | 0 ± 0 | 19.95 ± 7.95 | 24.75 ± 20 | 32.25 ± 7.5 | 92 ± 24 | 10.78 ± 1.53 | 4.25 ± 0.05 | 3.75 ± 0.75 | 4.18 ± 2.17 | 0.38 ± 0.38 | 0.58 ± 0.38 | 0.1 ± 0.1 | 0.65 ± 0.3 |
| Bivalvia | Mollusc larvae | 5.75 | 3.62 | 32.5 | 10.09 ± 4.91 | 6.29 ± 0.91 | 20.62 ± 7.62 | 1.88 ± 0.62 | 17 ± 2 | 5.38 ± 0.03 | 9.3 ± 2.8 | 19.55 ± 13.65 | 10.22 ± 8.53 | 0.15 ± 0.1 | 1.95 ± 0.15 | 1.2 ± 0 | 2.35 ± 1.75 |
