# Supplementary material for: Warming and trophic structure tightly control phytoplankton bloom amplitude, composition and succession
Source: PLoS One. 2024 Oct 4;19(10):e0308505. doi: 10.1371/journal.pone.0308505 (PMC11451980; doi:10.1371/journal.pone.0308505)
Supplement: Attachment — Submitted filename: report.docx [file pone.0308505.s003.docx]

In the manuscript “Warming and trophic structure tightly control phytoplankton bloom amplitude, composition and succession” the authors present the results of an experimental study investigating the effects of a temperature increase and the removal of large zooplankton on phytoplankton communities.

The study provides important insights into temperature's direct and indirect effects on trophic cascades. The most relevant contribution of this new scientific knowledge is the antagonistic interaction between the two manipulations on chlorophyll-a and how the treatments affect phytoplankton community composition. I made some comments for which I would like the authors to provide further information and clarifications.

Major comments:

1. I have two interrogations from a sentence in the introduction (L.71-72): The authors state, “There is a need to study the response of natural phytoplankton communities to warming, particularly in a top-down versus bottom-up context in more detail.”
   a. I understood this sentence to highlight the knowledge gap. Therefore, the study aims to distinguish the direct effect of temperature on phytoplankton from the indirect effect via the predator. The statistical analysis (see comment below) does not test the interaction.
   b. I would rephrase the sentence because the use of ‘versus’ suggests that temperature manipulation is a bottom-up control on phytoplankton (and directly refuted in the following sentence). The authors should clarify how the direct effect of temperature on phytoplankton can be distinguished from the indirect effect via predators.
2. The justification for excluding large zooplankton needs to be clearer. If increasing temperature might affect body size (and other life history traits), the larger zooplancton would not go extinct. The authors should strengthen this part of the introduction.
3. To answer their question regarding the two factors (temperature and large zooplankton), the authors analyzed their response variables with a 1-way ANOVA.
   a. However, a two-way ANOVA should be more appropriate, which will allow the authors to test and interpret the interaction between temperature and the large zooplankton exclusion. This result is one of the highlights of this study.
   b. Additionally, statistical information is too rare (except in Table 2 and the ‘stars’ on the figures). To strengthen the hard work of this study, at least the table (results from a 2-way RM-ANOVA for each period with the temporal interaction) for the chl-a and microzooplankton should appear in the main text (and the others in the supplementary information).
   c. By reading the results and looking at the figures and supplementary figures, it was unclear whether the RM-ANOVA was performed on each defined phase (pre-, post-, and bloom).

Minor comments:

Title. Warming and trophic structure tightly control phytoplankton bloom amplitude, composition and succession.

L24. “South of France”

L63-65. The plural “experimental studies” implies more than one study, so the authors should provide additional references.

L70. The reference [9] (Sommer and Lewandowska 2010) focused on zooplancton density rather than zooplancton size distribution. Therefore, this reference does not support the fact written in the sentence.

L70. I have a question regarding the sentence “suggesting that phytoplankton responses vary depending on phytoplankton size…” Should it be “zooplankton”?

L103. Did the pump bring oxygen to the system?

L103. What is the unit of the daily turnover time? If the water was renewed, was the filtering (1000µm and 200µm) also done?

L107-116. The authors should consider moving this paragraph to the “exclusion of mesozooplankton and increase in seawater temperatures” section.

L116-125. The authors should consider moving this paragraph to the “data/statistical analysis” section, as this information is important regarding the data analyzed.

L125. (and in all the text and figure legends). The “range of observation” is not precise enough. The authors should clarify it as standard deviation, standard error, or a 95% confidence interval. This information is useful for research reproducibility or meta-analysis.

L127. Consider removing “triplicate” because some have been removed from the study.

L138. Please format the reference Nouguier et al 2007 similarly to others.

L148. This sentence is confusing (especially the part “were extracted at these days to provide the missing temperature data”). Does it mean the authors used another method to replace the missing data from the multiparameter sensor? Could the authors simplify the sentence?

L161. What is the reference for the diffuse attenuation coefficient equation?

L176. Why were the mesozooplankton (>200µm) screened with a 20 µm net? Did the sampling occur in all mesocosms to check whether juveniles inoculated at the beginning of the experiment grew during the experiment?

L196. Should the concentration be another criterion to determine a bloom, not only the net growth?

L199. The authors do not discuss photoacclimation in the discussion; thus, they should add a reference here to support this statement.

L208. This sentence should reappear in the discussion section.

L228. What species have been identified as microprotozooplankton and micrometazooplankton?

L237-239. If the tintinnid abundance has been analyzed, the information needs to be presented in the results (there is no mention of tintinnid in the manuscript elsewhere).

L238. The sampling dates of the mesozooplankton do not correspond to the information in line 174.

L248. The effect of log-transformation should not be ‘assumed’ but checked… with the R package afex{}, a Greenhouse-Geiser option can be added to correct the lack of sphericity (similar to homoscedasticity for RM-ANOVA).

L300 (and others). The sentence “statistical differences of XXX in the different bloom phases identified with RM-ANOVA are presented in XXX” is confusing without a comma... does it mean the three phases were identified with the RM-ANOVA? Or were statistical differences detected with the RM-ANOVA?

L318. The sentence ends with “[…] with several exceptions.” Does the following sentence(s) explain these exceptions? Please make it clearer.

L403. Looking at Table S3, the removal of larger zooplankton does not seem efficient as the abundance of Acartia sp. (1 mm long) is similar between treatments, which agrees with the Authors’ statement in line 473. What could be the reason?

L464. The plural “previous studies” implies more than one study, so the authors should provide additional references.

L513. In the manuscript, the authors discriminated between micrometazooplankton and microprotozooplankton. Is it still the case in this sentence? (especially in the context of the next sentence).

Figure 8 is an excellent figure summarising the results!

L554-556. This statement must be tempered because the difference in the bloom is only one day in a mesocosm, so the difference might not be captured at a larger scale.
