# Supplementary material for: Warming and trophic structure tightly control phytoplankton bloom amplitude, composition and succession
Source: PLoS One. 2024 Oct 4;19(10):e0308505. doi: 10.1371/journal.pone.0308505 (PMC11451980; doi:10.1371/journal.pone.0308505)
Supplement: Attachment — Submitted filename: Response to Reviewers.docx [file pone.0308505.s004.docx]

Dear Academic Editor and Reviewers,

We would like to thank the Academic Editor and the two Reviewers for the additional comments, that significantly improved again this manuscript. We are grateful for their contributions.

Best wishes,

T. Trombetta, B. Mostajir, J. Courboulès, M. Protopapa, S. Mas, N. Aberle and F. Vidussi

Editor comments are in blue

Response to editor comments are in black with lines modified in the revised version with track changes indicated in parenthesis (LXXX).

LXXX: Indicated the lines and modifications made in the revised version WITH TRACK CHANGES., while words added in the revised version are underlined

**##################################################################################**

One reviewer pointed out that there are no references in the statistical treatment section and suggested you should add some references.

Response: References for statistical tests performed were added in the revised version (L251)

L251: To test the effect of the different treatments (C, MicroZ, T, and TMicroZ) two-way repeated measures analyses of variance (RM-ANOVA) [38] were conducted on the concentrations of nutrients, Chl *a*, and taxonomic pigments, as well as on pico- and nanophytoplankton abundances. Two factors of two levels (temperature: ambient temperature and elevated temperature Δ+ 3 °C; zooplankton community: natural planktonic community < 1000 µm and natural planktonic community < 200 µm) with interaction were tested and ‘days’ were set as random effect to prevent spurious autocorrelation effects. Normality was tested using the Shapiro-Wilk test and homogeneity of variance was assessed using the Levene test [39,40]. When assumptions were not meet despite logs, squared roots or Box-Cox transformations, Kruskall-Wallis tests were performed instead [41]. When significant, the comparison tests were followed by post hoc pairwise tests (Tukey’s tests for RM-ANOVA and Wilcoxon tests for Kruskall-Wallis) to identify the effects of the different treatments [42,43]. Comparison tests were conducted separately on the three different phytoplankton bloom periods observed during the experiment (‘pre-bloom’, ‘bloom’, and ‘post-bloom’; see the bloom period identification in the Result section) as effects were susceptible to inducing different responses on the various studied variables during these periods. As the bloom periods could have occurred at different times depending on the treatment, unified ‘pre-bloom’, ‘bloom’ and ‘post-bloom’ periods were identified using only the days of bloom period that were common among all treatments. As stated above, in this paper, we present two replicates of each treatment are presented and the “±” symbols in the text and related figures signify the range of the observations. Results of RM-ANOVA are presented in **S1 to S4 Fig**. To highlight shifts in phytoplankton community composition between periods and treatments, redundancy analysis (RDA) was performed [44]. ANOVA for RDA using the vegan package for R was then used to identify the environmental drivers of these community shifts. PERMANOVA were also conducted between treatments and periods to assess phytoplankton community composition differences, using Bray-Curtis distance on pigments and cytometry composition [45].

38. Muhammad LN. Guidelines for repeated measures statistical analysis approaches with basic science research considerations. J Clin Invest. 2023;133. doi:10.1172/JCI171058

39. Shapiro SS, Wilk MB. An Analysis of Variance Test for Normality (Complete Samples). Biometrika. 1965;52: 591–611. doi:10.2307/2333709

40. Brown MB, Forsythe AB. Robust Tests for the Equality of Variances. J Am Stat Assoc. 1974;69: 364–367. doi:10.2307/2285659

41. McKight PE, Najab J. Kruskal-Wallis Test. The Corsini Encyclopedia of Psychology. John Wiley & Sons, Ltd; 2010. pp. 1–1. doi:10.1002/9780470479216.corpsy0491

42. Rey D, Neuhäuser M. Wilcoxon-Signed-Rank Test. In: Lovric M, editor. International Encyclopedia of Statistical Science. Berlin, Heidelberg: Springer; 2011. pp. 1658–1659. doi:10.1007/978-3-642-04898-2_616

43. Keselman HJ, Rogan JC. The Tukey multiple comparison test: 1953–1976. Psychol Bull. 1977;84: 1050–1056. doi:10.1037/0033-2909.84.5.1050

44. van den Wollenberg AL. Redundancy analysis an alternative for canonical correlation analysis. Psychometrika. 1977;42: 207–219. doi:10.1007/BF02294050

45. Anderson MJ. Permutational Multivariate Analysis of Variance (PERMANOVA). Wiley StatsRef: Statistics Reference Online. John Wiley & Sons, Ltd; 2017. pp. 1–15. doi:10.1002/9781118445112.stat07841

I also noticed you did not state what software was used to carry out the statistical analysis, which should be stated for the benefit of the reader.

Response: The software used for statistical analysis was added in the revised version (L272)

L272: All statistical analysis were performed using R software version 4.4.0 (The R Foundation).
